# Seasonal variation of behavior and brain size in a freshwater fish

**DOI:** 10.1002/ece3.8179

**Published:** 2021-09-30

**Authors:** Evan J. Versteeg, Timothy Fernandes, Matthew M. Guzzo, Frédéric Laberge, Trevor Middel, Mark Ridgway, Bailey C. McMeans

**Affiliations:** ^1^ University of Toronto Mississauga Mississauga ON Canada; ^2^ Department of Integrative Biology University of Guelph Guelph ON Canada; ^3^ Harkness Laboratory of Fisheries Research Ontario Ministry of Natural Resources Whitney ON Canada

**Keywords:** acoustic telemetry, behavior, brain regions, brain size, habitat complexity, lake trout, phenotypic flexibility, seasonality

## Abstract

1. Teleost fishes occupy a range of ecosystem, and habitat types subject to large seasonal fluctuations. Temperate fishes, in particular, survive large seasonal shifts in temperature, light availability, and access to certain habitats. Mobile species such as lake trout (*Salvelinus namaycush*) can behaviorally respond to seasonal variation by shifting their habitat deeper and further offshore in response to warmer surface water temperatures during the summer. During cooler seasons, the use of more structurally complex nearshore zones by lake trout could increase cognitive demands and potentially result in a larger relative brain size during those periods. Yet, there is limited understanding of how such behavioral responses to a seasonally shifting environment might shape, or be shaped by, the nervous system.
2. Here, we quantified variation in relative brain size and the size of five externally visible brain regions in lake trout, across six consecutive seasons in two different lakes. Acoustic telemetry data from one of our study lakes were collected during the study period from a different subset of individuals and used to infer relationships between brain size and seasonal behaviors (habitat use and movement rate).
3. Our results indicated that lake trout relative brain size was larger in the fall and winter compared with the spring and summer in both lakes. Larger brains coincided with increased use of nearshore habitats and increased horizontal movement rates in the fall and winter based on acoustic telemetry. The telencephalon followed the same pattern as whole brain size, while the other brain regions (cerebellum, optic tectum, olfactory bulbs, and hypothalamus) were only smaller in the spring.
4. These findings provide evidence that flexibility in brain size could underpin shifts in behavior, which could potentially subserve functions associated with differential habitat use during cold and warm seasons and allow fish to succeed in seasonally variable environments.

## Graphical abstract

Seasonal variation in relative brain region size in lake trout. The curves show estimated marginal means and 95% confidence intervals (shaded ribbons) of telencephalon (a), cerebellum (b), optic tectum (c), olfactory bulbs (d), and hypothalamus (e) volumes. Letters denote significant seasonal differences obtained by Tukey's test.

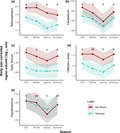

## 1 INTRODUCTION

Teleost fishes exhibit exceptional flexibility in their behavioral and physiological responses to changing environments, which have allowed them to colonize an impressive number of habitats at different latitudes (Armstrong & Bond, 2013; Dill, 1983). Previous work on freshwater fish has highlighted the need to better document and resolve the role of neural flexibility in underpinning life‐history strategies and ecology among different species and populations (Gonda et al., 2013). Fish often exhibit large variation in the proportional size of their brain regions in association with ecological and sensory specialization (Gonzalez‐Voyer & Kolm, 2010; Kotrschal et al., 1998). There is also a growing body of experimental literature, suggesting that novel environments can influence fish relative brain size within the span of a few weeks or months (Fong et al., 2019; Herczeg et al., 2015; Park et al., 2012; Turschwell & White, 2016; Závorka et al., 2020). If fish are capable of rapidly adjusting their brain size to cope with new environments, then perhaps fish can exhibit changes in brain size that allow them to succeed in the face of seasonal and interannual changes in natural environments.

Seasonality in temperate lake ecosystems generates dramatic declines in temperature and light levels during the late fall and winter due to shifting daylight cycles and ice/snow cover. Species time the phenology of many key life‐history events and activity patterns with these seasonal cycles. For example, while some warm water species suppress their activity in response to winter conditions (Shuter et al., 2012), cold‐adapted fish such as lake trout (*Salvelinus namaycush*) can be active all year round and exhibit marked seasonal changes in habitat use (Blanchfield et al., 2009). Specifically, during the summer months, when surface waters are warm, lake trout may reduce their activity levels and are largely restricted to cooler waters located deeper and further offshore in the lake (e.g., roughly when littoral water temperatures exceed 15°C; Martin, 1957, Guzzo et al., 2017). As temperatures decline, lake trout move nearshore to reproduce in the fall and can remain nearshore and actively swimming all winter (Blanchfield et al., 2009; McMeans et al., 2020). Such predictable seasonal shifts in activity levels and habitat use are commonplace among temperate fish species (e.g., to reach spawning sites or overwintering areas or to access prey; Hanson et al., 2008; Shuter et al., 2012) and mean that fish may experience differences in cognitive demands associated with reproduction or physical and visual complexity as they move between habitats (Pollen et al., 2007; Shumway, 2008). For instance, lake trout move from a more homogenous landscape of the pelagic environment during summer to a more structurally complex nearshore habitat often characterized by macrophytes and woody debris in the fall and winter (Caves et al., 2017; Shumway et al., 2007). Lake trout must also locate and capture prey under reduced lighting conditions during winter compared with other seasons (Blanchfield et al., 2009).

Fish brain size has been positively correlated with habitat complexity and habitat heterogeneity (Axelrod et al., 2018; Edmunds et al., 2016; Shumway, 2008) and is also correlated with performance in cognitively demanding tasks (Buechel et al., 2018; Kotrschal et al., 2013) and with variation in neuron numbers (Marhounová et al., 2019). As such, brain size variation is generally conceived as relating to variation in cognitive demands (i.e., sensory, motor, and integrative functions). Reproduction, sustained swimming, and navigation in nearshore environments might therefore be expected to increase cognitive demands during fall and winter compared with spring and summer. To date, however, only a single study has explored seasonal variation in the relative size of one brain region (the telencephalon) in a wild fish, the round goby (*Neogobius melanostomus*). This study found relatively larger telencephalon sizes during the reproductive season, which was associated with the increased spatial processing demands of mating (McCallum et al., 2014). It is still unknown, however, whether whole brain size is seasonally flexible in fishes, or whether the size of individual brain regions can change independently of one another across seasons.

**FIGURE 1:**
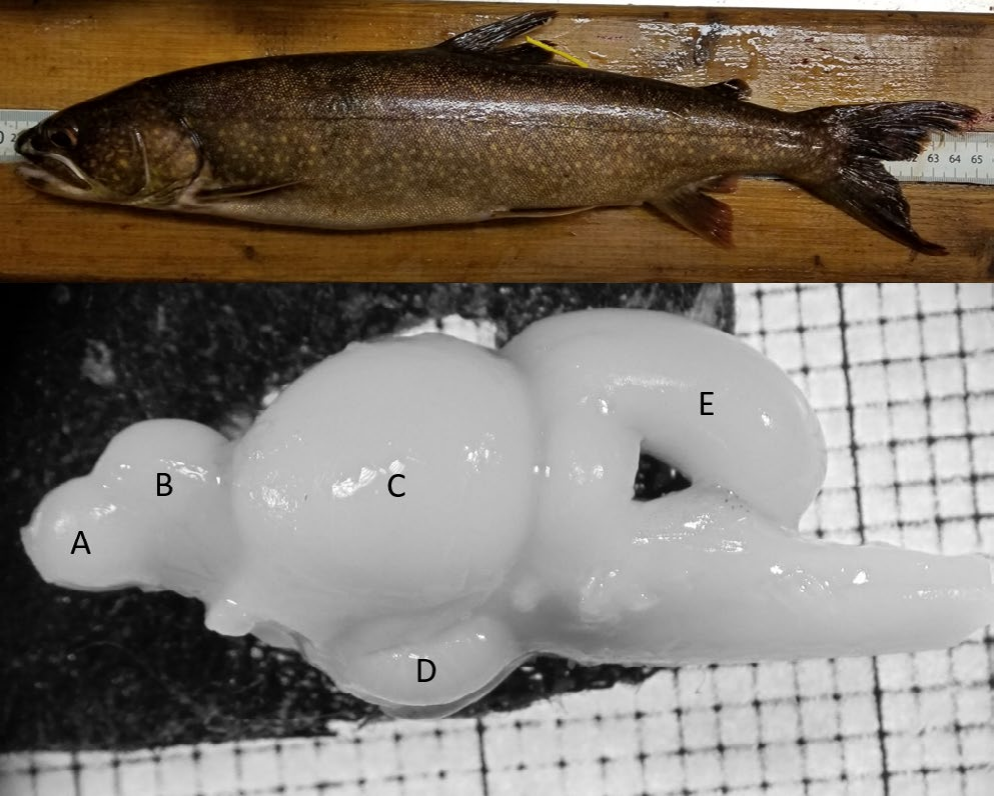
Lake trout (top) and a lake trout brain with letters indicating the regions measured in the present study (bottom). The olfactory bulbs (a) process olfactory information. The telencephalon (b) is primarily associated with spatial navigation and learning. The optic tectum (c) processes visual and multimodal sensory information used for orientation. The cerebellum (d) is associated with motor coordination and learning. The hypothalamus (E) is involved in behavioral and neuroendocrine control (Huber et al., 1997; Pollen et al., 2007)

Here, we investigated the seasonal variability in the relative size of the brain and some of its constituent regions (Figure 1) in lake trout (*Salvelinus namaycush*) from two lakes in Ontario, Canada. We first hypothesized that seasonal changes in cognitive demands would drive changes in lake trout relative brain size over a seasonal timescale. For example, as lake trout are nearshore during the fall and winter, its structurally complex habitat along with heightened lake trout activity may necessitate relatively larger brains compared with offshore habitat use in the spring and summer. Second, we hypothesized that any changes in relative brain size were either the result of: (a) seasonal demands for region‐specific processing (i.e., a mosaic change in the size of some brain regions and not others), as certain regions might be of more or less utility depending on season‐specific life history, or (b) ubiquitous changes in the relative size of each brain region (i.e., concerted change in brain size; Finlay & Darlington, 1995; Striedter, 2005). Brain size data were collected in both lakes over six consecutive seasons. Trends in brain size variation were compared with habitat use and movement rate data obtained by acoustic telemetry from one of our study lakes to assess the relationship between seasonal patterns in brain size and behavior.

## 2 MATERIALS AND METHODS

### 2.1 Study sites and sample collection

Lake trout were collected seasonally from Lake of Two Rivers (hereafter referred to as “Two Rivers”), Ontario, Canada (45^o^34’42.6” N, 78^o^29’0.4” W; 274 ha surface area, 38 m maximum depth), and Lake Opeongo (hereafter referred to as “Opeongo”), Ontario, Canada (45^o^41’46.8” N, 78^o^22’27.8” W; 5,800 ha surface area, 49.4 m maximum depth), each located within Algonquin Provincial Park. Unlike Two Rivers, Opeongo supports pelagic prey fish (e.g., lake cisco: *Coregonus artedi*). Mature lake trout were sampled seasonally from fall 2017 to winter 2019 (Table 1) using trap nets, gill nets, and angling equipment. The fish were euthanized immediately upon capture via severing of the spinal cord, and their lengths and weights collected (under approved University of Toronto Animal Use Protocols). Fish heads were then removed and placed in labeled containers with 10% neutral‐buffered formalin (Fisher Scientific Inc., New Jersey, USA). Fish that possessed undeveloped gonads were considered immature and removed from analysis.

**TABLE 1:** Sample sizes of lake trout for each season (*n*) and the corresponding date range within which they were sampled from lakes Opeongo and Two Rivers, Ontario

Lake	Season	Sample Period	Temp. °C	*n*
Opeongo	Fall 2017	17 October–2 November	12	9
	Winter 2018	13–16 March	Ice cover	12
	Spring 2018	11–17 May	4	16
	Summer 2018	31 August–10 September	20	22
	Fall 2018	30 October–2 November	10	14
	Winter 2019	18–21 March	Ice cover	11
Two Rivers	Fall 2017	30 October	11	6
	Winter 2018	19–21 March	Ice cover	6
	Spring 2018	8–9 May	4˚	20
	Summer 2018	20–21 August, 12–15 September	23, 19	17
	Fall 2018	30 October–1 November	6	21
	Winter 2019	12–15 March	Ice cover	6

Note

The mean daily surface water temperature (<6 m depth) is provided unless the loggers had been removed due to ice cover.

Following previous conventions (Guzzo et al., 2017), the period after ice‐off but before mean surface temperature (<6 m depth) exceeded 15˚C was denoted as spring, summer was defined as the period during which surface temperatures reached or surpassed 15˚C, and fall began when lakes cooled to ≤15˚C and lasted until winter, defined as ice‐on to ice‐off. Water temperatures were measured throughout the upper 6 m of the water column in each lake using a string of data loggers (HOBO Temp Pro H20‐001, Onset, Cape Cod, MA) deployed over the deepest point of each lake (Table 1).

### 2.2 Brain mass and region volumes

Following procedures from Edmunds et al. (2016), the brains were removed and trimmed of cranial nerves, and the spinal cord was cut at the obex. Each brain was blotted thoroughly to remove excess formalin and was then weighed on an analytical balance (Fisher Scientific Accu‐124D), to a resolution of 0.0001 g to obtain brain mass, which was used to estimate brain size. Pictures were taken of the dorsal, left, and ventral sides of the brain using an Olympus SZ61 dissection microscope and a Canon Powershot G9 digital camera and PSREMOTE v1.7 software (Breeze & Breeze, 2009). A calibration grid was included in each picture. The height, length, and width of brain regions visible on the resulting images (olfactory bulbs, telencephalon, optic tectum, cerebellum, and hypothalamus) were measured using the measuring tool in ImageJ software (Rueden et al., 1997). Regional volumes (mm^3^) were estimated using the ellipsoid formula: *V* = (*L* × *W* × *H*) *π*/6, providing us with estimates of size for each brain region. Volume measurements were conducted by the same person (EJV), and measurement accuracy was assessed by repeating measurements of five randomly selected brains ten times (Appendix S1: Table S1).

### 2.3 Acoustic telemetry

Acoustic telemetry data were available from Lake of Two Rivers throughout the study period, albeit for a different subset of lake trout individuals (*n* = 9). Fixed location reference tags within the array provided a measure of array performance throughout the study period. Estimated positions based on the Vemco (InnovaSea, WA, USA) positioning algorithm were compared with known positions of the tags to quantify positioning errors across seasons and years using methods proposed in Smith (2013). Reference tags at 5 m depth had a lower average error than tags placed at 18 m depth. Over 95% of detections at 5 m had a positional error of less than 6 m vs. 86% at the 18 m depth. Overall mean error was 2.41 m vs 5.24 m at 5 and 18 m depth, respectively. Additional details about the telemetry array setup, performance, and fish tagging can be found in Appendix S2.

### 2.4 Statistical analysis

**FIGURE 2:**
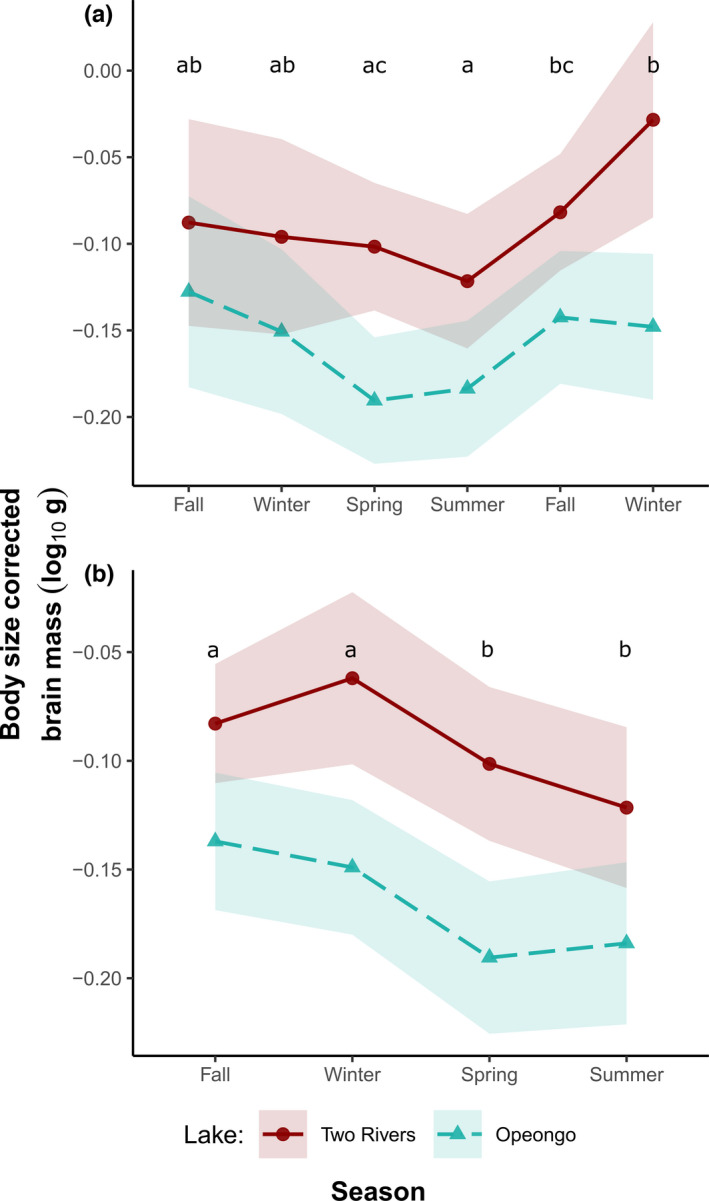
Seasonal variation in relative brain size in lake trout. The curves show estimated marginal means and 95% confidence intervals (shaded ribbons) of brain mass corrected for fork length in trout from Lake of Two Rivers (red, solid line) and Lake Opeongo (blue, dashed line). (a) Model of relative brain size variation across six consecutive seasons. (b) Model combining two fall and winter sampling seasons. Different letters denote significant seasonal differences (*p* < .05) obtained by Tukey's test

Seasonal differences in lake trout brain mass and each brain region volume were explored using separate linear models (LM’s). Log_10_‐transformed brain mass or brain region volume was the dependent variable; season, lake, and sex were included as fixed factors; and Log_10_‐transformed fork length was included as a covariate (fork length was positively linearly related to brain mass and the volume of the individual regions; Appendix S1: Table S2 and Figure S1). An alpha of 0.05 was used for all analyses. An interaction between lake and season was included to evaluate whether the effect of season on brain size varied between lakes. Preliminary analyses indicated that the main effect of sex and the interaction between sex and season did not significantly influence the models, so sex was removed from the final analysis. Similarly, if the main effect of sex was not a significant parameter for a given model, it was removed. Following a significant main effect of season, seasonal differences were identified using Tukey's pairwise comparisons of estimated marginal means (EMMs). The initial model for brain mass was run including all six seasons spanning our two study years (Table 1). After finding no significant differences between the two fall seasons or the two winter seasons (Figure 2a), these seasons were combined (resulting in 4 seasons: spring, summer, fall, winter) in all subsequent analyses of brain mass and individual brain regions.

Acoustic telemetry data were modeled using generalized additive mixed models (GAMMs; Wood, 2011, 2017). After filtering the data (Appendix S3), we performed four separate analyses on: (1) weekly mean depth, (2) standard deviation (SD) of depth, (3) mean distance to shore, and (4) mean horizontal movement rate (Appendix S1: Table S5). We fit two models for each of these response variables. The first model, known as the year‐specific smoother model (YS), had separate seasonal smoothers for each year (March 2017–March 2018 and March 2018–March 2019) by including a factor–smoother interaction between week of the year and year, with seasonality in behavior represented by week of the year and treated as a cyclic cubic regression spline (knots = weeks 1–52). The second model, known as the common smoother model, contained a single seasonal smoother treated as a cyclic cubic regression spline common to both years of data. In each model, we included fish ID as a random effect, and autoregressive and moving average (ARMA) correlation structures were added to the models to account for autocorrelation. The correlation structures used in each model, where p is the order of the autoregressive (AR) part and q is the order of the moving average (MA), were *p* = 2, *q* = 1 for mean depth, SD depth, and mean distance to shore and *p* = 2, *q* = 2 for movement rate. Once the most appropriate correlation structures were identified for each response variable, we fit the two candidate models using maximum likelihood and ranked them using AIC. The model with the lowest AIC value was chosen and a difference in AIC < 2 meant equal support for both models, in which case the simpler CS model was chosen. We then refit the selected model using restricted maximum likelihood to obtain parameter estimates and significance (*α* = 0.05).

## 3 RESULTS

### 3.1 Hypothesis 1: brain size

When including data for all six seasons sampled across our two study years, Opeongo lake trout relative brain size decreased from fall and winter highs to spring and summer lows before increasing again in the fall and winter of the second study year (Figure 2a). In Two Rivers, larger relative brain size in fall and winter was limited to the second year of sampling (Figure 2a). Season and lake had significant effects on lake trout relative brain size according to the LM, though there was no significant season–lake interaction (Table 2). Fall and winter of the first year did not significantly differ from fall and winter of the second year (Figure 2a, Appendix S1: Table S3), and the data from each of these seasons over two years were combined for subsequent analyses (resulting in 4 seasons).

**TABLE 2:** Results of linear models exploring the main effects of season, lake, and the season‐lake interaction on total brain mass and the volumes of individual regions (telencephalon, cerebellum, optic tectum, olfactory bulb, and hypothalamus)

Variable	Season	Lake	Season*Lake
Brain mass (continuous)	*p* < .001	*p* < .001	*p* = .180
	*F*_5,128_ = 13.7	*F*_1,127_ = 0.86	*F*_5,120_=1.55
Brain mass (combined)	*p* = .003	*p* < .001	*p* = .32
	*F*_3,130_ = 5.00	*F*_1,129_ = 97.73	*F*_3,127_ = 1.26
Telencephalon volume	*p* < .001	*p* < .001	*p* = .311
	*F*_3, 130_ = 8.80	*F*_1, 129_ = 17.2	*F*_3, 124_ = 1.2
Cerebellum volume	*p* < .001	*p* < .001	*p* = .850
	*F*_3, 130_ = 10.5	*F*_1, 129_ = 99.9	*F*_3, 124_ = 0.27
Optic tectum volume	*p* = .017	*p* < .001	*p* = .785
	*F*_3, 130_ = 3.55	*F*_1, 129_ = 76.1	*F*_3, 124_ = 0.36
Olfactory bulb volume	*p* < .001	*p* = .001	*p* = .619
	*F*_3, 130_ = 6.09	*F*_1, 129_ = 10.8	*F*_3, 124_ = 0.600
Hypothalamus volume	*p* = .007	*p* < .001	*p* = .715
	*F*_3, 130_ = 4.17	*F*_1, 129_ = 85.8	*F*_3, 124_ = 0.45

Note

Changes in brain mass were first explored across all six continuous seasons (i.e., fall, winter, spring, summer, fall, winter) before combining data into 4 seasons (i.e., fall, winter, spring, summer) for subsequent comparisons of brain mass and regional volumes.

In the LM performed on the four seasons, season and lake had significant effects on lake trout relative brain size (Table 2). Opeongo trout: (a) were larger than Two Rivers trout (mean fork length ± SD: 510 ± 54.6 vs. 410 ± 40.2 mm; mean body mass ± SD: 1574 ± 504 vs. 775 ± 228 g), (b) had a larger mean absolute brain size (0.832 ± 0.134 SD vs. 0.692 ± 0.134 g), but (c) had a smaller relative brain size compared with Two Rivers trout (i.e., after correcting for body size; Appendix S1: Table S2 and Figure S1). The effect of season on relative brain size did not differ between lakes (season–lake interaction was not significant; Table 2). Tukey's pairwise comparisons of seasonal EMMs demonstrated that relative brain size was smaller during the spring and summer and larger during the fall and winter when the two study years were combined (Figure 2b, Appendix S1: Table S3).

### 3.2 Hypothesis 2: brain region sizes

Next, we evaluated whether changes in brain size were region‐specific or whether changes involved all regions observable at the gross morphology level. When using a LM with fork length as a covariate to correct for variation in body size, the main effect of lake was significant: Fish from Two Rivers had relatively larger telencephala (lake: F_2, 134_ = 250, *p* < .001), cerebella (lake: F_2, 134_ = 260, *p* < .001), optic tecta (lake: F_2,134_ = 370.6, *p* > .001), and olfactory bulbs (lake: F_2,134_ = 205.4, *p* > .001) than those from Opeongo. There was a significant interaction between fork length and lake for hypothalamus size (F_3, 133_ = 109, *p* = .003), where different allometric slopes produced Two Rivers fish with larger hypothalami than Opeongo fish at smaller fork lengths (Appendix S1: Figure S1).

**FIGURE 3:**
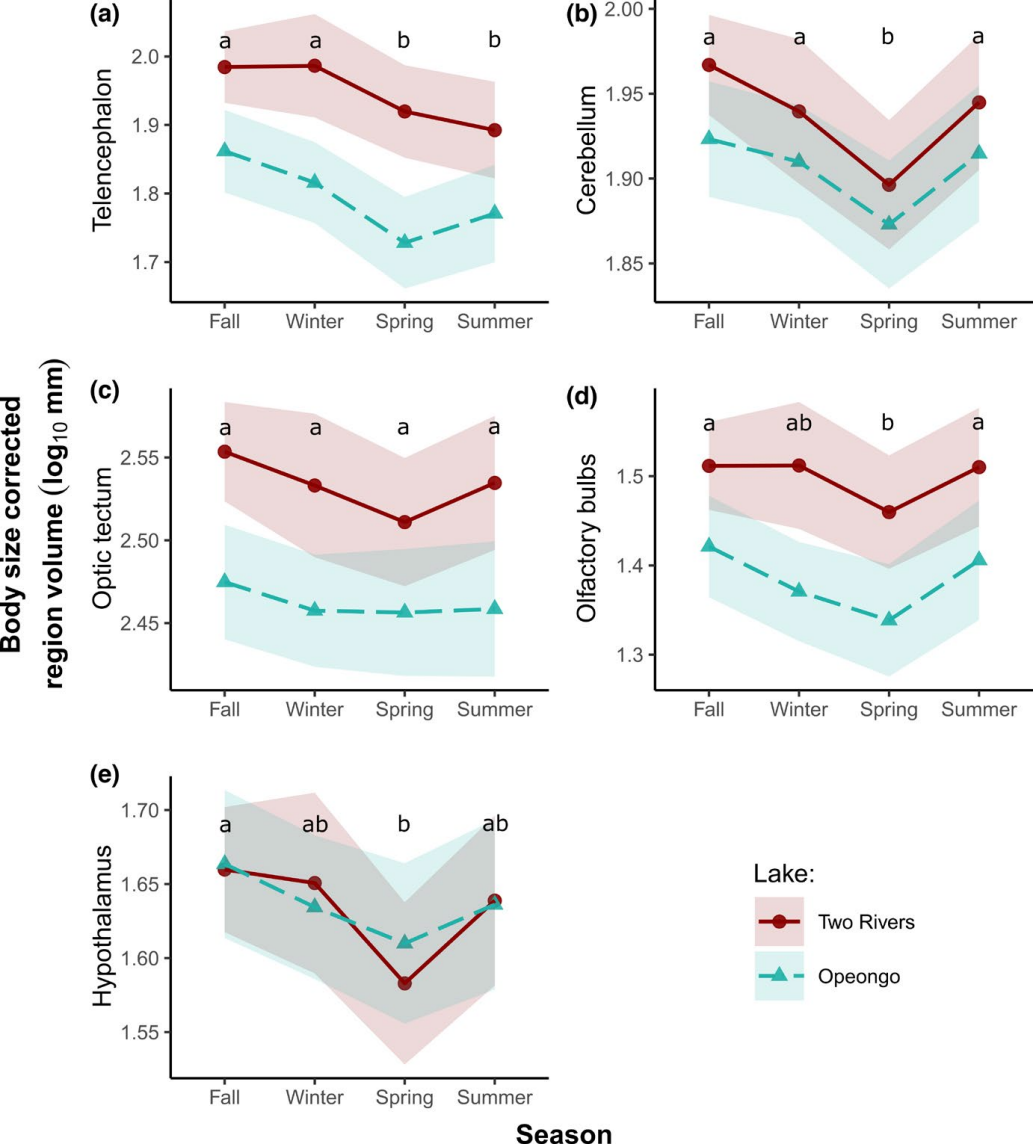
Seasonal variation in relative brain region size in lake trout. The curves show estimated marginal means and 95% confidence intervals (shaded ribbons) of telencephalon (a), cerebellum (b), optic tectum (c), olfactory bulbs (d), and hypothalamus (e) volumes corrected for fork length in trout of Lake of Two Rivers (red, solid line) and Lake Opeongo (blue, dashed line). Different letters denote significant seasonal differences (*p* < .05) obtained by Tukey's test of models combining two fall and winter sampling seasons

The main effect of season and lake was also significant for all five brain regions, yet none of the regions demonstrated a significant interaction between season and lake. Thus, the seasonal patterns observed in brain regions were not population‐specific (Table 2). Telencephalon size followed a trend similar to that of the whole brain (smaller in spring and summer, larger in fall and winter; Figure 3a), while cerebellum size was smallest during the spring, with no significant differences between the fall, winter, and summer (Figure 3b, Appendix S1: Table S3). Although season was a significant factor in the LM, the optic tecum did not differ between any seasons based on the Tukey tests (*p* > .06; Appendix S1: Table S4); however, the general trend in this region was for lower size in the spring (Figure 3c). Like the cerebellum, both the olfactory bulbs and the hypothalamus were smallest during the spring, with no significant difference between summer, fall, and winter (Figure 3d, e, Appendix S1: Table S4).

### 3.3 Acoustic telemetry

**FIGURE 4:**
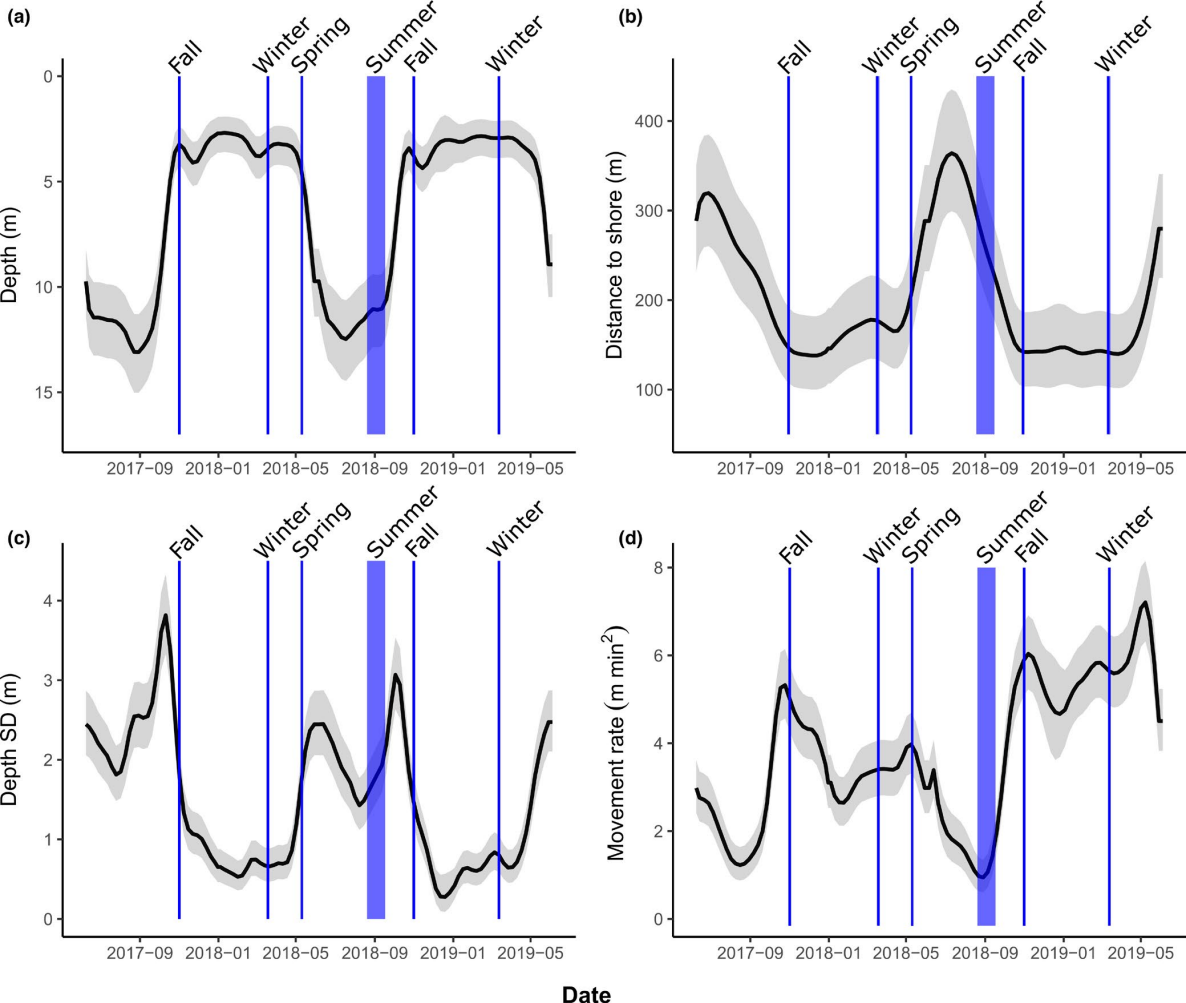
Seasonal variation in lake trout behavior assessed by telemetry. The weekly mean depth (a), distance to shore (b), standard deviation (SD) of depth (i.e., vertical activity; c), and movement rates (d) are shown. The trout were tagged with acoustic tags and tracked using 3D telemetry during 9 June 2017–7 June 2019 in Lake of Two Rivers, Ontario. The lines represent mean fits, and ribbons are the 95% CI estimated using generalized additive mixed effects models with year‐specific cyclic cubic regression spline smoothers. Vertical lines represent the sampling periods of trout used to measure brain size. The thicker blue line for summer represents a longer sampling period during that season (dates provided in Table 1)

Model selection indicated that each of the movement and habitat use metrics from Lake of Two Rivers trout was best modeled using a GAMM with a year‐specific weekly smoother rather than a common weekly smoother (Appendix S1: Table S4). During the summer, lake trout occupied greater depths (~12 m; Figure 4a) and were located well over 300 meters away from shore (Figure 4b). Pronounced vertical movement in the water column was characteristic of summer months (high SD of depth; Figure 4c), while horizontal movements were lowest at this time (Figure 4d). Conversely, lake trout occupied shallower depths during the coolest weeks of the year (fall, winter, early spring; Figure 4a). Rapid depth changes occurred around weeks 20 and 40 (mid‐May and early October), with fish moving offshore to deeper water in the late spring (immediately following the Spring sample collected in May) and back nearshore into shallow water in early October (Figure 4a). Fish remained in generally shallow water throughout the winter. These periods of low depth occupancy in the spring, fall, and winter coincided with shorter distances from shore (Figure 4b) and a low SD in depth (Figure 4c), indicating that the fish were not regularly changing position in the water column during that time. Horizontal movement rates were lowest in the summer and increased dramatically in the fall (Figure 4c). Horizontal movement rates were higher in the second compared with the first winter; however, this did not seem to be due to differences in system positioning error between winters.

## 4 DISCUSSION

Our data suggest that the brain size of a temperate freshwater fish changes within an annual timespan, mirroring seasonal changes in habitat use and movement. Two Rivers lake trout also consistently possessed relatively larger brains than Opeongo lake trout. Lake of Two Rivers is smaller in size and more circular in shape than Lake Opeongo. Unlike Opeongo, Two Rivers also lacks an offshore forage fish. Together, these lake characteristics should be associated with Two Rivers lake trout foraging more frequently in complex nearshore areas (Dolson et al., 2009; Vander Zanden & Rasmussen, 1996), which is consistent with previous observations that fish species and populations with a higher reliance on the nearshore habitat have larger brains (Axelrod et al., 2018; Edmunds, Laberge, et al., 2016). Despite these differences, seasonal variation in relative brain size was apparent across both lakes, with brains being smallest during the spring and summer and largest during the fall and winter. It is unlikely that lake temperature alone was responsible for these trends. Though larger brain sizes have been associated with higher temperatures (and possibly metabolism; Gillooly & McCoy, 2014; Yu et al., 2014; Závorka et al., 2020), we observed that lake trout relative brain size was smallest during the warmest season (summer). Previous work has suggested that increases in brain size reflect increased performance in cognitively demanding tasks (Buechel et al., 2018; Kotrschal et al., 2013). Our findings therefore support our first hypothesis that fish might alter their brain size seasonally, potentially in accordance with seasonal variation in cognitive demands.

Variation in whole brain size reflects underlying variation in individual brain regions, which were also found to vary seasonally. The telencephalon tracked the observed changes in brain size best (larger in the fall and winter compared with spring and summer). Larger telencephala have been associated with higher utilization of nearshore habitats (Edmunds, McCann, et al., 2016; Gonzalez‐Voyer & Kolm, 2010). In their investigations of the round goby *Neogobius melanostomus*, McCallum et al. (2014) also found larger telencephalon sizes prior to the spawning season. As lake trout move nearshore onto spawning shoals in the fall, they navigate more structurally heterogeneous environments, while also coordinating complex social behaviors during mating (Johnson et al., 2018). Nearshore habitat use was maintained throughout the winter (at least in telemetered Two Rivers lake trout), which would also be associated with navigating under reduced light conditions (due to reduced photoperiod and snow and ice cover; Blanchfield et al., 2009). Larger telencephala in the fall and winter could therefore reflect increased cognitive demands associated with spawning in the fall and nearshore habitat use during the fall and winter. Nearshore foraging is also known to occur in spring (Guzzo et al., 2017) however, and telemetered lake trout in our study had just begun to move away from shore at the time of spring sampling. Smaller telencephala in the spring and summer could, therefore, be associated with movement into offshore, deeper water. Additional analysis of the telencephalon revealed that this region was larger in the fall than the spring when accounting for variations in brain size (i.e., using brain size as a covariate instead of body size), suggesting that this region fluctuates independently of seasonal fluctuation in overall brain size (Figure S2).

Unlike the telencephalon, seasonal changes in size of the other brain regions were limited to a decrease in size in the spring. Nearshore habitat use is higher in fall and winter compared with summer, and reproduction occurs in the fall (Guzzo et al., 2017). Habitat use and spawning alone are likely insufficient to explain the size of these brain regions, which were of similar size in fall, summer, and winter. The smaller size of the cerebellum, olfactory bulb, optic tectum, and hypothalamus in spring vs. fall may potentially be driven by increased lake trout foraging and growth during the spring (Fry, 1939; Guzzo et al., 2017). Large increases in total body length during spring months, for example, may possibly reduce brain size relative to body size; such a dilution would be expected whether brain growth lags behind patterns in somatic growth. Alternatively, reduced relative brain sizes during the spring may be a product of energy trade‐off mechanisms that mediate energetically expensive tissues to optimize growth potential. Trade‐offs in brain and gut size, for example, have been observed in fish and other vertebrates (Aiello & Wheeler, 1995; Kotrschal et al., 2013; Monnet et al., 2020; Rosenfeld et al., 2020). Investment in large, energetically expensive digestive organs in support of growth in the spring could entail temporary reduction in brain size to allow a more effective allocation of resources to different parts of the organism (Armstrong & Bond, 2013). Whether such organ system trade‐offs happen on a seasonal scale is unknown. Future work is required to explore how seasonally changing cognitive and energetic demands might govern the size of the brain and its regions in lake trout, and to determine whether brain size changes are the result of variation in neuron numbers (Marhounová et al., 2019) or fluctuations in body size.”

Previous work has identified seasonal brain size flexibility. The size of the telencephalic hemispheres and whole brain of a benthic fish and a shrew, respectively, have exhibited distinct seasonal patterning (Lázaro et al., 2018; McCallum et al., 2014). Seasonal variation in the size of a specific brain region, the hippocampus, has also been noted in birds and mammals (Yaskin, 2011). However, the inability to record brain metrics from the same individuals over time represents a central limitation to our study (and previous studies on this topic), given that, at this time, animals must be sacrificed to measure brain sizes.

We therefore cannot discount the possibility that individuals captured during fall and winter tended to be those individuals within the population that had relatively larger brains. We corrected for variation in body size among captured individuals and attempted to avoid sampling bias by using both gill netting and angling to capture lake trout during all seasons. However, winter‐caught fish were predominantly captured via angling due to the logistical difficulty associated with gill netting under ice (i.e., the net remained in a single location and was not moved around from location to location as was the case during open water sampling). Gill nets were most successful during open water seasons for capturing lake trout. Previous work suggests that both gill netting and angling catch similarly sized lake trout with similar behaviors and diets (Luo et al., 2019), and in our study, lake trout from both fall and winter had relatively larger brains despite being captured predominately by different equipment (gill nets and angling, respectively). We have also included a limited set of ecological traits (i.e., habitat use, movement rates, fall reproduction by using mature individuals), making our inferences about the role of brain size in these behaviors tentative until a larger suite of traits can be explored. Additionally, we echo previous concerns about the uncertain role of brain size in cognition and stress caution when interpreting its relationship with complex behaviors (Healy & Rowe, 2007). However, the fact that we observed the same seasonal trend in both lakes and the observation that larger relative brain size coincided with increased nearshore habitat use and movement rates in telemetered Lake of Two Rivers lake trout suggest that brain size of lake trout is seasonally flexible in support of, or in response to, changes in behavior. More work will be needed to elucidate which specific behaviors are impacted by seasonally flexible changes in brain size.

## 5 CONCLUSIONS

Here, we find novel evidence that brain size changes seasonally in two wild populations of a top‐predator teleost fish. The whole brain and telencephalon sizes were smaller in spring and summer, while the other brains regions investigated were only smaller in the spring. These distinct seasonal cycles could subserve different functions associated with differential habitat use during cold and warm seasons, and differential energetic allocation of resources between tissues in support of foraging and growth in the spring. Further investigation will be needed to understand the mechanisms that drive seasonal variation in brain size and to identify the specific roles that this phenomenon plays in the regulation of fish behavior and physiology.

## CONFLICT OF INTEREST

None declared.

## AUTHOR CONTRIBUTIONS

**Evan J. Versteeg:** Data curation (equal); Formal analysis (lead); Investigation (lead); Methodology (equal); Writing‐original draft (lead); Writing‐review & editing (lead). **Timothy Fernandes:** Data curation (equal); Investigation (supporting); Methodology (lead); Writing‐original draft (equal); Writing‐review & editing (supporting). **Matthew M. Guzzo:** Formal analysis (equal); Investigation (equal); Methodology (supporting); Writing‐review & editing (supporting). **Frederic Laberge:** Conceptualization (lead); Data curation (equal); Funding acquisition (equal); Methodology (equal); Project administration (supporting); Resources (lead); Writing‐review & editing (equal). **Trevor Middel:** Data curation (equal); Project administration (supporting); Resources (lead); Writing‐review & editing (supporting). **Mark Ridgway:** Data curation (equal); Methodology (equal); Resources (supporting); Writing‐review & editing (supporting). **Bailey C. McMeans:** Conceptualization (lead); Data curation (supporting); Funding acquisition (lead); Methodology (equal); Project administration (lead); Resources (lead); Writing‐original draft (supporting); Writing‐review & editing (equal).

## Supporting information

Appendix S1

Appendix S2

Appendix S3

## Data Availability

Data and code are available through Dryad Digital Repository: https://10.5061/dryad.0cfxpnw35.
