# Supplementary material for: Seasonal variation of behavior and brain size in a freshwater fish
Source: Ecol Evol. 2021 Sep 30;11(21):14950–9. doi: 10.1002/ece3.8179 (PMC8571637; doi:10.1002/ece3.8179)
Supplement: Supplementary file 1 — Appendix S1 [file ECE3-11-14950-s003.docx]

**Supporting Information.** **Seasonal variation of behavior and brain size in a freshwater fish**

**Appendix S1.**

Table S1. Coefficients of Variation (CV) of five repeated volume measurements for each of the following brain regions: the telencephalon, cerebellum, optic tectum, olfactory bulbs, and hypothalamus. Ten repeated measurements were conducted for five different brains (listed as Brain 1-5), and then the CV’s for each region was averaged across the five brains to provide a final estimate of CV for each brain region. CV was calculated as standard deviation/mean x 100.

|  | Region Volumes Coefficients of Variation (%) | | | | |
| --- | --- | --- | --- | --- | --- |
| Sample | Telencephalon | Cerebellum | Optic Tectum | Olfactory Bulbs | Hypothalamus |
| Brain 1 | 2.68 | 3.43 | 2.07 | 6.94 | 7.19 |
| Brain 2 | 1.74 | 1.83 | 1.73 | 6.54 | 4.98 |
| Brain 3 | 1.88 | 12.08 | 13.44 | 3.61 | 18.17 |
| Brain 4 | 4.06 | 1.92 | 2.73 | 7.53 | 14.41 |
| Brain 5 | 26.32 | 25.27 | 26.54 | 20.52 | 27.31 |
| Average | 7.34 | 8.91 | 9.30 | 9.03 | 14.41 |

Table S2. Lake-specific linear regression equations of the relationships of log_10_ brain mass (g) and log_10_ brain region volumes (mm^3^) as functions of fish log_10_ fork length (mm).

| Variable | Lake | Equation | P | R^2^_adj_ |
| --- | --- | --- | --- | --- |
| Brain mass | Opeongo | log_10_ mass = 1.64 x log_10_ fork length - 4.54 | <0.001 | 0.77 |
|  | Two Rivers | log_10_ mass = 1.55 x log_10_ fork length - 4.24 | <0.001 | 0.71 |
| Telencephalon | Opeongo | log_10_ volume = 1.58 x log_10_ brain mass + 2.04 | <0.001 | 0.91 |
|  | Two Rivers | log_10_ volume = 1.42 x log_10_ brain mass + 2.08 | <0.001 | 0.88 |
| Cerebellum | Opeongo | log_10_ volume = 0.86 x log_10_ brain mass + 2.04 | <0.001 | 0.83 |
|  | Two Rivers | log_10_ volume = 0.75 x log_10_ brain mass + 2.01 | <0.001 | 0.76 |
| Optic tectum | Opeongo | log_10_ volume = 0.96 x log_10_ brain mass + 2.62 | <0.001 | 0.86 |
|  | Two Rivers | log_10_ volume = 0.87 x log_10_ brain mass + 2.61 | <0.001 | 0.84 |
| Olfactory bulb | Opeongo | log_10_ volume = 1.15 x log_10_ brain mass + 1.56 | <0.001 | 0.74 |
|  | Two Rivers | log_10_ volume = 1.10 x log_10_ brain mass + 1.60 | <0.001 | 0.73 |
| Hypothalamus | Opeongo | log_10_ volume = 0.88 x log_10_ brain mass + 1.77 | <0.001 | 0.75 |
|  | Two Rivers | log_10_ volume = 0.69 x log_10_ brain mass + 1.70 | <0.001 | 0.39 |

Table S3. Estimated linear contrasts (Estimate), standard errors (SE), Z-test statistics, and P-values for comparisons of brain size between seasons. Contrasts were conducted on: 1) continuous seasons: across all six seasons of Fall 2017 (Fall 1), Winter 2018 (Winter 1), Spring, Summer, Fall 2018 (Fall 2), Winter 2019 (Winter 2), and 2) on combined seasons: after combining the seasons of the two study years (Fall, Winter, Spring, Summer).

| Continuous Seasons | | | | |
| --- | --- | --- | --- | --- |
| Contrast | Estimate | SE | Z-statistic | P-Value |
| Fall 1 – Winter 1 | 0.01563 | 0.0195 | 0.803 | 0.9671 |
| Fall 1 – Spring | 0.03838 | 0.0170 | 2.255 | 0.2130 |
| Fall 1 – Summer | 0.04490 | 0.0169 | 2.651 | 0.0855 |
| Fall 1 – Fall 2 | 0.00443 | 0.0171 | 0.259 | 0.9998 |
| Fall 1 – Winter 2 | -0.01955 | 0.0190 | -1.030 | 0.9080 |
| Winter 1 – Spring | 0.02274 | 0.0153 | 1.487 | 0.6725 |
| Winter 1 – Summer | 0.02927 | 0.0154 | 1.899 | 0.4029 |
| Winter 1 – Fall 2 | -0.01120 | 0.0150 | -0.748 | 0.9759 |
| Winter 1 – Winter 2 | -0.03518 | 0.0173 | -2.038 | 0.3207 |
| Spring – Summer | 0.00652 | 0.0123 | 0.531 | 0.9949 |
| Spring – Fall 2 | -0.03394 | 0.0120 | -2.838 | 0.0516 |
| Spring – Winter 2 | -0.05793 | 0.0146 | -3.961 | **0.0011** |
| Summer – Fall 2 | -0.04047 | 0.0119 | -3.393 | **0.0090** |
| Summer – Winter 2 | -0.06445 | 0.0145 | -4.437 | **0.0001** |
| Fall 2 – Winter 2 | -0.02398 | 0.0143 | -1.681 | 0.5441 |
| Combined Seasons | | | | |
| Contrast | Estimate | SE | Z-statistic | P-value |
| Fall-Winter | -0.00445 | 0.0111 | -0.401 | 0.9782 |
| Fall-Spring | 0.03600 | 0.0112 | 3.207 | **0.0073** |
| Fall-Summer | 0.04277 | 0.0112 | 3.826 | **0.0008** |
| Winter-Spring | 0.04046 | 0.0123 | 3.282 | **0.0057** |
| Winter-Summer | 0.04722 | 0.0123 | 3.830 | **0.0007** |
| Spring-Summer | 0.00676 | 0.0124 | 0.544 | 0.9481 |

Table S4. Estimated linear contrasts (Estimate), standard errors (SE), Z-test statistics, and P-values for comparisons of brain region size between seasons. Contrasts were conducted across the four seasons after combining the seasons of the two study years (Fall, Winter, Spring, Summer).

| Contrast | Estimate | SE | Z-statistic | P-value |
| --- | --- | --- | --- | --- |
| Telencephalon | | | | |
| Fall-Winter | 0.02200 | 0.0212 | 1.039 | 0.7263 |
| Fall-Spring | 0.09920 | 0.0214 | 4.636 | **<.0001** |
| Fall-Summer | 0.09149 | 0.0213 | 4.294 | **0.0001** |
| Winter-Spring | 0.07720 | 0.0235 | 3.285 | **0.0056** |
| Winter-Summer | 0.06948 | 0.0235 | 2.956 | **0.0164** |
| Spring-Summer | -0.00771 | 0.0237 | -0.326 | 0.9881 |
| Cerebellum | | | | |
| Fall-Winter | 0.02037 | 0.0120 | 1.703 | 0.3218 |
| Fall-Spring | 0.06049 | 0.0121 | 5.005 | **<.0001** |
| Fall-Summer | 0.01532 | 0.0120 | 1.273 | 0.5802 |
| Winter-Spring | 0.04012 | 0.0133 | 3.023 | **0.0134** |
| Winter-Summer | -0.00505 | 0.0133 | -0.380 | 0.9813 |
| Spring-Summer | -0.04517 | 0.0134 | -3.377 | **0.0041** |
| Optic Tectum | | | | |
| Fall-Winter | 0.01885 | 0.0122 | 1.550 | 0.4076 |
| Fall-Spring | 0.03048 | 0.0123 | 2.480 | 0.0630 |
| Fall-Summer | 0.01756 | 0.0122 | 1.435 | 0.4775 |
| Winter-Spring | 0.01163 | 0.0135 | 0.862 | 0.8245 |
| Winter-Summer | -0.00129 | 0.0135 | -0.096 | 0.9997 |
| Spring-Summer | -0.01292 | 0.0136 | -0.950 | 0.7778 |
| Olfactory Bulb | | | | |
| Fall-Winter | 0.02496 | 0.0200 | 1.251 | 0.5943 |
| Fall-Spring | 0.06722 | 0.0202 | 3.333 | **0.0048** |
| Fall-Summer | 0.00838 | 0.0201 | 0.417 | 0.9756 |
| Winter-Spring | 0.04226 | 0.0221 | 1.908 | 0.2246 |
| Winter-Summer | -0.01658 | 0.0222 | -0.749 | 0.8773 |
| Spring-Summer | -0.05884 | 0.0223 | -2.636 | **0.0418** |
| Hypothalamus | | | | |
| Fall-Winter | 0.01914 | 0.0173 | 1.104 | 0.6871 |
| Fall-Spring | 0.06528 | 0.0176 | 3.717 | **0.0012** |
| Fall-Summer | 0.02421 | 0.0173 | 1.397 | 0.5012 |
| Winter-Spring | 0.04614 | 0.0192 | 2.407 | 0.0757 |
| Winter-Summer | 0.00507 | 0.0191 | 0.266 | 0.9934 |
| Spring-Summer | -0.04107 | 0.0192 | -2.136 | 0.1418 |

Table S5. Model selection based on Akaike information criterion with a correction for small sample sizes (AICc) for generalized additive mixed models modelling seasonal variation of movement and habitat use of lake trout tracked using 3D acoustic telemetry in Lake of Two Rivers, Ontario during June 9, 2017 - June 7, 2019. Model selection compared the fit of a year-specific smoother (YS) and a common smoother (CS) which combined both years. Shaded rows highlight the model smoother with the lowest AICc value.

| Response | Model | logLik | AICc | ∆AICc | df | Deviance  explained | K | Wi |
| --- | --- | --- | --- | --- | --- | --- | --- | --- |
| Mean depth (m) | YS | -412.0 | 910.9 | 0.0 | 43.5 | 0.80 | 52 | 0.997 |
|  | CS | -432.2 | 923.2 | 12.2 | 29.4 | 0.80 | 52 | 0.002 |
| SD of depth (m) | YS | -41.6 | 169.6 | 0.0 | 43.2 | 0.67 | 52 | 1.000 |
|  | CS | -70.9 | 201.2 | 31.6 | 29.8 | 0.65 | 52 | <0.001 |
| Mean distance to shore  (m) | YS | -2026.4 | 4107.1 | 0.0 | 27.1 | 0.62 | 52 | 0.998 |
|  | CS | -2039.1 | 4119.7 | 12.6 | 20.7 | 0.61 | 52 | 0.002 |
| Mean movement rate  (m min^-1^) | YS | -297.2 | 671.0 | 0.0 | 38.3 | 0.65 | 52 | 1.000 |
|  | CS | -434.9 | 917.9 | 246.9 | 24.0 | 0.51 | 52 | <0.001 |


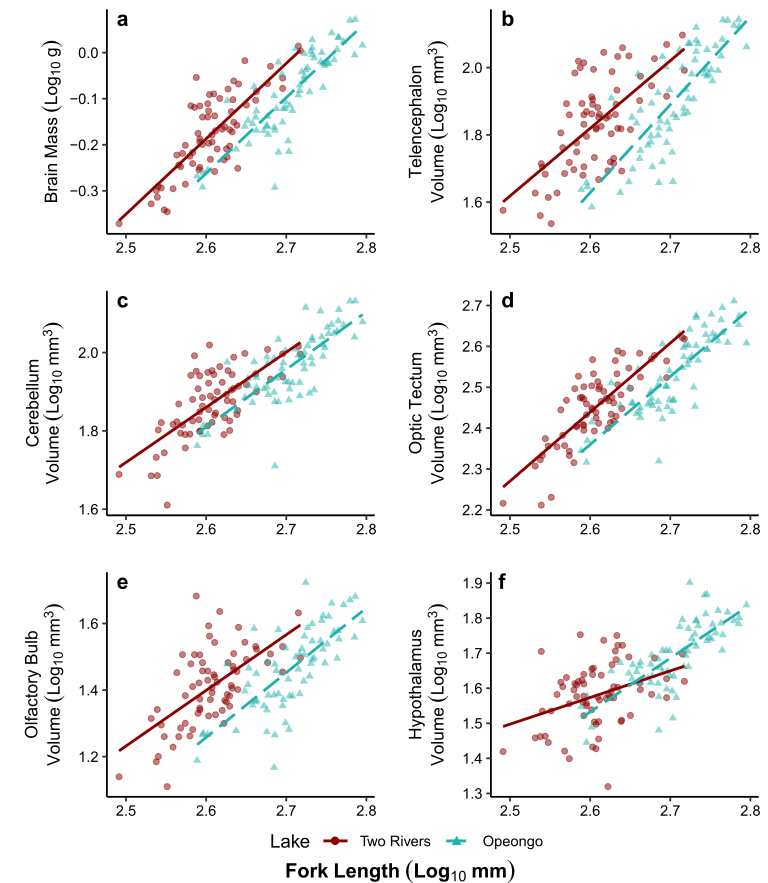


Figure S1. Lake-specific linear regressions between log_10_ brain mass and log_10_ fork length (A) and log_10_ brain region volume and log_10_ fork length (B: telencephalon; C: cerebellum; D: optic tectum; E: olfactory bulbs, F: hypothalamus) in lake trout collected from Lake of Two Rivers (red, solid line and circles) and Lake Opeongo (blue, dashed line and triangles), Ontario, Canada.

**
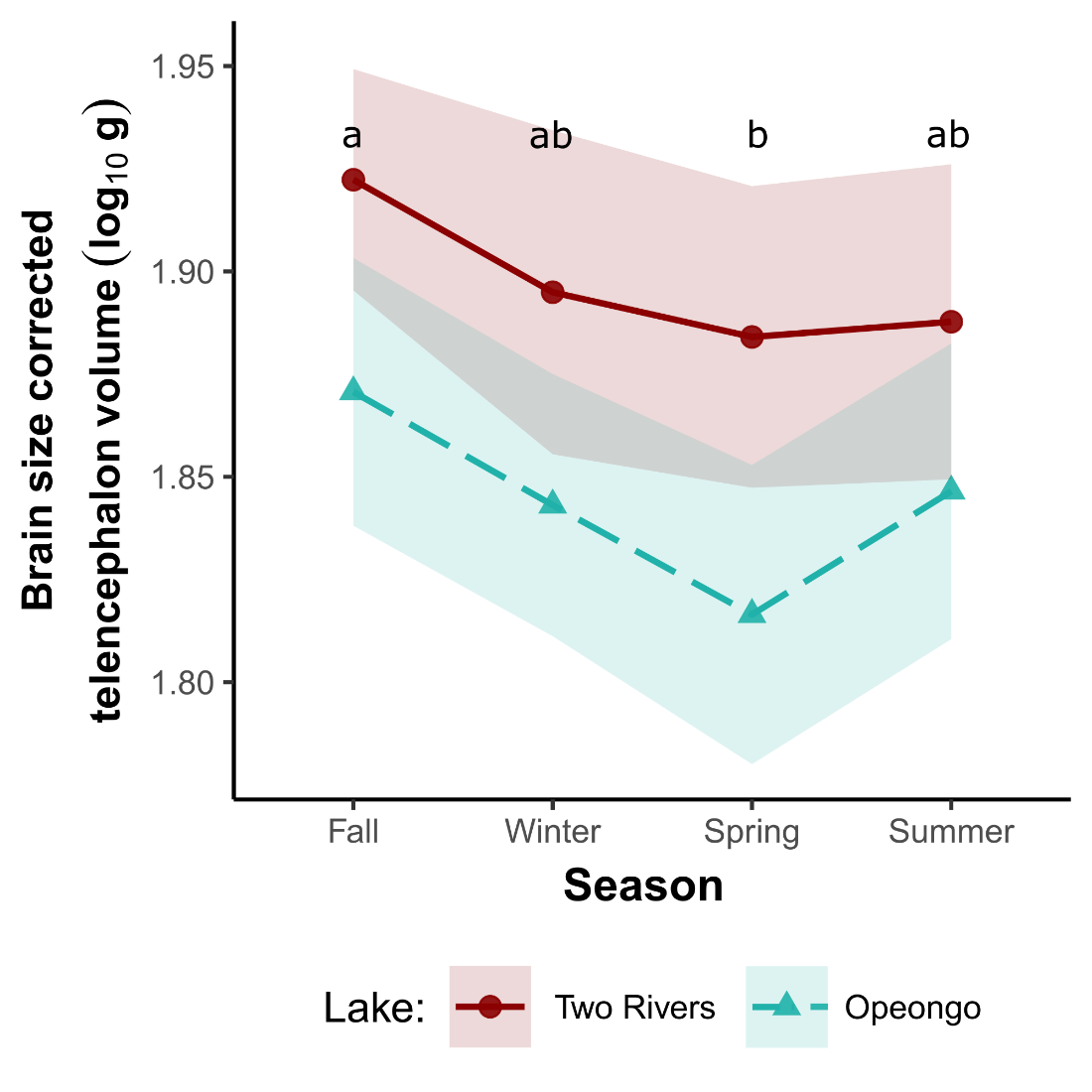
**

Figure S2. Seasonal variation of lake trout telencephalon size relative to brain mass. The curves show estimated marginal means and 95% confidence intervals (shaded ribbons) corrected for brain mass in trout of Lake of Two Rivers (red, solid line) and Lake Opeongo (blue, dashed line). Different letters denote significant seasonal differences (P < 0.05) obtained by Tukey’s test of models combining two fall and winter sampling seasons.
