# Supplementary material for: Seasonal variation of behavior and brain size in a freshwater fish
Source: Ecol Evol. 2021 Sep 30;11(21):14950–9. doi: 10.1002/ece3.8179 (PMC8571637; doi:10.1002/ece3.8179)
Supplement: Supplementary file 2 — Appendix S2 [file ECE3-11-14950-s002.docx]

**Supporting Information.** **Seasonal variation of brain size in a freshwater top predator**

**Appendix S2.**

*Acoustic telemetry setup –* The Lake of Two Rivers telemetry array consists of 54 omnidirectional receivers (69 kHz VR2W, Vemco Ltd., Bedford, NS, Canada) with co-located sync tags (V16, Vemco Ltd). The mean distance between receivers was 236 m. Receivers were affixed at a depth of 2 m to vertical lines anchored to two 8 kg steel weights and suspended by a surface float. A synchronization tag was also affixed to each line below the receiver. Floats were submerged beneath the lake surface in the fall to protect from the effects of ice over winter. Six reference tags (V9P) were deployed throughout the array at depths of 5 m and 18 m to provide fixed positions which could be used to evaluate the performance of the array. Tags implanted in lake trout were pressure sensing V9P (Vemco Ltd, Bedford, NS, Canada) tags programmed to transmit at a pseudo-random interval, on average every 420 s, ranging between 360 and 480 s. Estimated tag life was 912 days. The V9P tags were 9 mm X 31 mm and weighed 2.8 g in water. Surgeries to implant tags into lake trout were performed over three periods, May 2017, September 2017, and May 2018. During each of these periods, lake trout were captured either by angling, trap net, or short set duration gillnet. Upon capture, if fish were deemed to be in good condition, they were anaesthetized using a buffered solution of tricaine methanesulfonate (MS-222), placed into a wetted foam surgery cradle where a flow of maintenance dose buffered MS-222 solution was directed across their gills. A small incision was made midline and posterior to the pectoral fins using a clean surgical scalpel and the telemetry tag was inserted. The incision was stitched with 2-3 sutures using 3-0 Monocryl synthetic absorbable sutures (Ethicon Inc, Somerville, NJ, USA). Length (±1 mm) and scale samples were obtained from the fish and an external t-tag was applied at the base of the dorsal fin so the fish could be identified if captured by an angler or during future sampling. The total time for surgery and sampling was typically less than 3 min per fish. The fish was then placed into a tank filled with lake water and allowed to recover until it was able to right itself and exhibited strong tailbeats, which typically occurred within 10-15 min. The fish was then returned to the lake. Receivers were downloaded every six months; as the receiver retrieval, download, and redeployment process took several days, there are periods in the spring and fall for which detection data are not used given the array was in a state of flux. Raw detection data were processed by Vemco using a hyperbolic positioning algorithm (Smith, 2013) to assign x, y positions to fish detected at multiple receivers over the course of the study. In this study we used data from 9 individual lake trout with data spanning the entire study period (June 9 2017 - June 7 2019), resulting in a total of 382,829 raw detections (18,415- 59,168 per fish).

**Literature Cited**

Smith, F. (2013). Understanding HPE in the VEMCO Positioning System (VPS). In *Understanding HPE in the VEMCO Positioning System (VPS)* (pp. 1–31). https://doi.org/VEMCO Document # Doc-005457-07
