# Supplementary material for: Seasonal variation of behavior and brain size in a freshwater fish
Source: Ecol Evol. 2021 Sep 30;11(21):14950–9. doi: 10.1002/ece3.8179 (PMC8571637; doi:10.1002/ece3.8179)
Supplement: Supplementary file 3 — Appendix S3 [file ECE3-11-14950-s001.docx]

**Supporting Information.** **Seasonal variation of brain size in a freshwater top predator**

**Appendix S3.**

*Acoustic Telemetry Analysis -* We filtered the telemetry data. First, data collected in the first 14 days following tag implantation were omitted from analyses to minimize potential alterations to behavior caused by surgery (Rogers & White, 2007). Next, we created a depth dataset by removing positions that fell outside the boundary of the lake or had depths above the water surface (0 m) or below the maximum depth of the lake (27 m) from the raw dataset. This depth dataset included a total of 382,725 depth detections (range 18,415-59,075 per fish). We then used the depth dataset to calculate the weekly mean depth and standard deviation (SD) of depth of each individual lake trout. The SD of depth for each period was used as a proxy for variation in depth use or vertical activity (Freitas et al., 2015; Skilbrei & Otterå, 2016). Next, we created a dataset to calculate the distance to the nearest shoreline for each fish position. For this distance to shore dataset, we started with the raw dataset and removed all positions that fell outside the boundary of the lake, giving a total of 378,523 spatial positions (range 18,333-57,666 per fish). We then used this dataset to calculate the weekly mean distance to shore of each individual lake trout. Finally, to estimate movement rates, we took the distance to shore dataset and calculated the displacement in meters and difference in timestamp in minutes between all successive positions for each fish. We then divided the spatial displacement by the timestamp difference to get a movement rate (m min^-1^) between all successive positions for each fish throughout the study. We then filtered out movement rates estimates that were based on successive positions detected >20 min apart or that were >40 m min^-1^, which we considered as a conservative estimate of maximum swimming speed based on the results of (cruz-font et al 2019). This resulted in a dataset of 293,723 movement rates (range 11,220-47,877 per fish) with an average difference in timestamp of 9.27 min (mean range per fish 9.07-9.82 min). We then used this movement rate dataset to calculate the weekly mean movement rate of each individual lake trout.

**Literature Cited**

Freitas, C., Olsen, E. M., Moland, E., Ciannelli, L., & Knutsen, H. (2015). Behavioral responses of Atlantic cod to sea temperature changes. *Ecology and Evolution*, *5*(10), 2070–2083. https://doi.org/10.1002/ece3.1496

Rogers, K. B., & White, G. C. (2007). Analysis of movement and habitat use from telemetry data. In *Analysis and interpretation of freshwater fisheries data* (pp. 625–676). http://cpw.state.co.us/Documents/Research/Aquatic/pdf/Publications/AnalysisOfMovementAndHabitat.pdf

Skilbrei, O. T., & Otterå, H. (2016). Vertical distribution of saithe (Pollachius virens) aggregating around fish farms. *ICES Journal of Marine Science*, *73*(4), 1186–1195. https://doi.org/10.1093/icesjms/fsv261
